# 血管免疫母细胞性T细胞淋巴瘤伴单克隆B细胞、浆细胞增生

**DOI:** 10.3760/cma.j.issn.0253-2727.2023.09.015

**Published:** 2023-09

**Authors:** 文强 严, 婧钰 许, 灵纳 李, 瑞 吕, 黎红 寿, 琦 孙, 慧君 王, 录贵 邱, 刚 安

**Affiliations:** 1 中国医学科学院血液病医院（中国医学科学院血液学研究所），实验血液学国家重点实验室，国家血液系统疾病临床医学研究中心，细胞生态海河实验室，天津 300020 State Key Laboratory of Experimental Hematology, National Clinical Research Center for Blood Diseases, Haihe Laboratory of Cell Ecosystem, Institute of Hematology & Blood Diseases Hospital, Chinese Academy of Medical Sciences & Peking Union Medical College, Tianjin 300020, China; 2 天津医学健康研究院，天津 301600 Tianjin Institutes of Health Science, Tianjin 301600, China; 3 湖州市中心医院血液科，湖州 313000 Department of Hematology, Huzhou Central Hospital, Huzhou 313000, China

患者，男，57岁，因“发现全身多处淋巴结肿大半年余，乏力消瘦2个月余”于2022年6月21日入院。患者半年余前无明显诱因出现发热，体温最高达39 °C，伴咽痛，伴颈部、腋下、腹股沟多发淋巴结肿大，无压痛，自行服用药物后可退热，反复发热3 d就诊于当地医院，查血常规：WBC 7.7×10^9^/L，HGB 123 g/L，PLT 165×10^9^/L；EB病毒（EBV）DNA：2.77×10^3^/L；浅表淋巴结超声：双侧颈部、腹股沟多发淋巴结肿大；PET-CT：横膈上下多发肿大淋巴结，氟代脱氧葡萄糖（FDG）代谢增高（SUVmax＝15.79）；脾大（SUVmax＝4.08）。患者2021年12月2日于外院行左侧锁骨上淋巴结切检，病理会诊符合EBV相关性淋巴组织增生性病变，建议密切随访观察，观察期间出现双腿一过性片状红斑及瘙痒。2022年3月7日复查血常规：WBC 5.7×10^9^/L，HGB 130 g/L，PLT 113×10^9^/L，外周血涂片可见2％异型淋巴细胞，外院予干扰素α2a 135 µg皮下注射2次，后逐渐出现乏力、纳差、发热、周身水肿，伴双下肢一过性皮疹，伴恶心、呕吐，伴尿量减少，乏力呈进行性加重，2022年4月急查血肌酐1 030.4 µmol/L；24 h尿蛋白2.86 g；血、尿免疫固定电泳κ轻链阳性；骨髓涂片可见42％浆细胞，以淋巴样浆细胞为主；骨髓流式细胞术：可见异常B细胞6.953％，异常浆细胞11.535％；骨髓病理：造血组织增生尚活跃（35％），浆细胞片状分布，考虑浆细胞增生性病变。为明确病因行肾穿刺活检：肾活检组织改变以急性重度肾小管损伤为主，同时存在间质弥漫浸润细胞，肾小球病变较轻，经免疫电镜检查证实存在轻链近端肾小管病（LCPT），但不能排除其他因素（如药物）引起急性肾小管损伤可能。综合考虑为急性肾衰竭，行血液透析数次、甲泼尼龙治疗后肌酐逐渐降至300 µmol/L，尿量逐渐恢复。2022年4月为明确诊断再次行左侧颈深淋巴结切除术，病理会诊考虑符合浆细胞肿瘤；淋巴结流式细胞术：异常浆细胞占19.0％。复查骨髓穿刺：淋巴样浆细胞28％，易见异常淋巴浆细胞。B细胞淋巴瘤基因突变提示：TNFRSF14突变41.5％，该基因突变可能常见于滤泡性淋巴瘤；MYD88（−）。2022年5月复查PET-CT：横膈上下多发肿大淋巴结，部分融合，较前大致相似（SUVmax＝10.82）；脾大，FDG代谢增高（SUVmax＝4.32）；全身骨髓FDG代谢弥漫性增高；考虑浆细胞性淋巴瘤浸润所致。两肺多发多形性病变，FDG代谢未见增高，考虑淋巴瘤浸润可能性大。外院考虑淋巴瘤，暂予对症支持治疗，患者乏力、纳差无明显缓解，为进一步诊治就诊于中国医学科学院血液病医院。患者自发病以来，精神欠佳，食欲明显减退，睡眠可，大小便正常，体重下降12 kg，2013年因胃恶性肿瘤行胃切除术。

入院查体：慢性病容，营养不良，中度贫血貌。双下肢可见片状红斑，无黄染、出血点，颈部双侧、腹股沟可触及肿大浅表淋巴结，最大者约4 cm×4 cm，无压痛，活动度可。肝肋缘下未触及，脾肋缘下可触及。双下肢轻度水肿，神经系统检查未见异常。

血常规：WBC 5.04×10^9^/L，HGB 72 g/L，PLT 78×10^9^/L；肝肾功能：总蛋白58.5 g/L，白蛋白24.3 g/L，肌酐340.6 µmol/L，尿素10.09 mmol/L，乳酸脱氢酶157.2 U/L；血清蛋白电泳：可见一条单克隆轻链κ成分；M片段：17.22％，M蛋白10.07 g；血β_2_-微球蛋白19.8 mg/L；血清游离轻链比值1.21；24 h尿蛋白11.28 g；尿M片段98.78％，尿M蛋白11.14 g；血尿免疫固定电泳均可见单克隆轻链κ成分。血清EBV-DNA<1 000拷贝/ml；B细胞：32 301拷贝/ml；T细胞：2 440拷贝/ml；NK细胞：23 372拷贝/ml。心肌损伤标志物无异常；细菌真菌标志物无异常。全身CT：颈部、胸部、上腹部、盆腔多发淋巴结，部分淋巴结肿大；双肺感染性病变；心包增厚，左侧少量胸腔积液，腹腔、盆腔积液，颈部、胸壁、上腹部、盆腔皮下水肿。

2022年6月22日行骨髓穿刺示：此部位骨髓增生重度减低，可见25％浆细胞，易见浆样淋巴细胞骨髓象；外周血涂片正常。骨髓活检病理：HE及PAS染色示送检骨髓增生较低下（约20％），浆细胞增多（约40％），散在或簇状分布，形态轻度异型，淋巴细胞散在分布，胞体小，各阶段粒红系细胞散在分布，巨核细胞少见。网状纤维染色（MF-0级）。免疫组化：CD20个别细胞（+），CD3少数（+），CD5少数（+），CD4少数（+），CD8散在（+），CD10（−），CD138浆细胞（+），κ浆细胞（+），λ浆细胞（−），CyclinD1（−）。诊断：骨髓增生较低下，单克隆浆细胞增生，未见异型淋巴细胞明显增多。染色体核型：46，XY[20]。染色体荧光原位杂交：CEP7/D7S486缺失阴性；MYC基因重排阴性；TP53/CEP17缺失阴性；MYB基因未见异常。融合基因：IGK、IGH、IGL重排阳性；TCRγ、TCRβ重排阳性。

## 第一次临床讨论

患者中老年男性，以多发淋巴结增大起病，反复发热，伴乏力、纳差，伴大量蛋白尿，且出现一过性急性肾衰竭，血尿可见异常M蛋白，EBV-DNA明显升高，外院淋巴结活检病理考虑EBV驱动的淋巴细胞恶性增殖合并浆细胞疾病，骨髓流式细胞术可见三群异常细胞（单克隆浆细胞、B细胞、T细胞），需与以下疾病鉴别：

1. 多发性骨髓瘤（MM）：是以浆细胞恶性增殖为特征的恶性疾病，好发于中老年人，男性多见，以多发溶骨性破坏、贫血、高钙血症、肾功能损害为典型表现，多数患者伴有异常M蛋白升高，骨髓可见大量异常浆细胞增生。本例患者为中老年男性，血尿中可见κ轻链，且出现一过性肾衰竭，经皮肾脏活检术提示轻链肾小管病，骨髓穿刺可见大量单克隆浆细胞增生，但该患者多发淋巴结增大且反复发热，PET-CT并未提示骨质破坏，不符合MM典型表现，但仍应考虑MM可能。

2. 淋巴浆细胞淋巴瘤/华氏巨球蛋白血症（LPL/WM）：是一种较为少见的惰性成熟B细胞淋巴瘤，好发于老年人，常侵犯骨髓、淋巴结和脾脏，大部分患者诊断时有肿瘤浸润症状（贫血、发热、盗汗、体重减轻、淋巴结肿大、肝脾大），伴血清单克隆IgM增高或高黏滞综合征，分子遗传学可有MYD88阳性。本例患者可见多发淋巴结增大，伴发热，且骨髓中可见单克隆浆细胞、小B淋巴细胞浸润，但MYD88阴性且无IgM升高，需进一步除外非典型LPL/WM可能。

3. 血管免疫母细胞性T细胞淋巴瘤（AITL）：占外周T细胞淋巴瘤（PTCL）的15％～20％，以男性多见，主要表现为B症状（发热、盗汗和体重减轻）和淋巴结增大，超过70％出现骨髓浸润，而肝脾大少见，多数患者确诊时已处于晚期，发病与EBV高度相关，起源于表达CXCL13、CD10和PD-1的滤泡辅助T细胞（TFH），病理见滤泡树突状细胞（FDC）和分枝样高内皮小静脉（HEV）增生是AITL的主要特点。本例患者以多发淋巴结增大起病，伴反复发热，后出现消瘦和双下肢一过性皮疹，伴多浆膜腔积液，符合AITL典型临床表现。同时该患者起病时查血清EBV-DNA水平明显升高，B细胞、NK细胞EBV-DNA升高明显，但外院淋巴结病理活检未见典型AITL表现及TFH标志，骨髓可见大量单克隆浆细胞，伴大量蛋白尿、急性肾衰竭等，二代测序未见典型重现性基因突变，尚无确凿证据，需进一步病理会诊并加做免疫组化以辅助诊断。

4. POEMS综合征：是一种少见的、独立的单克隆浆细胞疾病，发病年龄为40～60岁，男性多于女性，发病机制尚不清楚，临床上以多发性周围神经病、脏器肿大、内分泌障碍、M蛋白血症和皮肤病变为特征，常伴血管内皮生长因子（VEGF）升高，骨髓可见单克隆浆细胞增生。该患者可见单克隆浆细胞增殖，血、尿异常M蛋白，多发淋巴结肿大，双下肢一过性皮疹，但并无多发性周围神经病变、硬化性骨病、Castleman病等表现，暂不符合POEMS综合征诊断，可进一步完善VEGF、肌电图等除外。

5. EBV相关淋巴组织增殖性疾病（LPD）：并非一种独立的疾病，主要可分为EBV^+^B细胞LPD和EBV^+^T/NK细胞LPD两大类。本例患者以淋巴结肿大为突出表现，且外院淋巴结活检提示EBER^+^活化B细胞，但该患者病程迁延，出现发热、乏力、消瘦、急性肾衰竭等症状，不符合典型EBV^+^LPD。

## 第二次临床讨论

回顾2022年6月骨髓穿刺流式细胞术结果，可见三群异常细胞（图1）：①异常T淋巴细胞占5.47％，表达CD2、CD4、CD5、CD45RO、PD-1，不表达CD3、CD8、CD7、CD10、CD30、CD16、CD56、CD57；CD3^−^CD7^−^提示为典型异常T淋巴细胞，而CD4^+^CD10^−^PD-1^++^CD26^dim^CD45RO^+^，且TCR重排阳性，符合AITL典型表型；②成熟单克隆小B淋巴细胞占4.44％，表达CD19、FMC7、CD22、CD20、CD79b、CD81、κ，不表达CD5、CD10、CD23、CD103、CD25、CD11c、sIgD、CD38、CD200、sIgM、λ，不符合常见B细胞LPD典型表型，考虑为AITL相关支持性细胞及炎性背景细胞可能性大；③单克隆浆细胞占28.65％，强表达CD38、CD138，表达CD19、CD27、CD81、cκ，弱表达CD45，不表达CD56、CD117、CD20、CD200、CD28、cλ，为正常浆细胞表型，呈cκ单克隆，并不符合MM肿瘤细胞典型免疫表型。

**图1:**
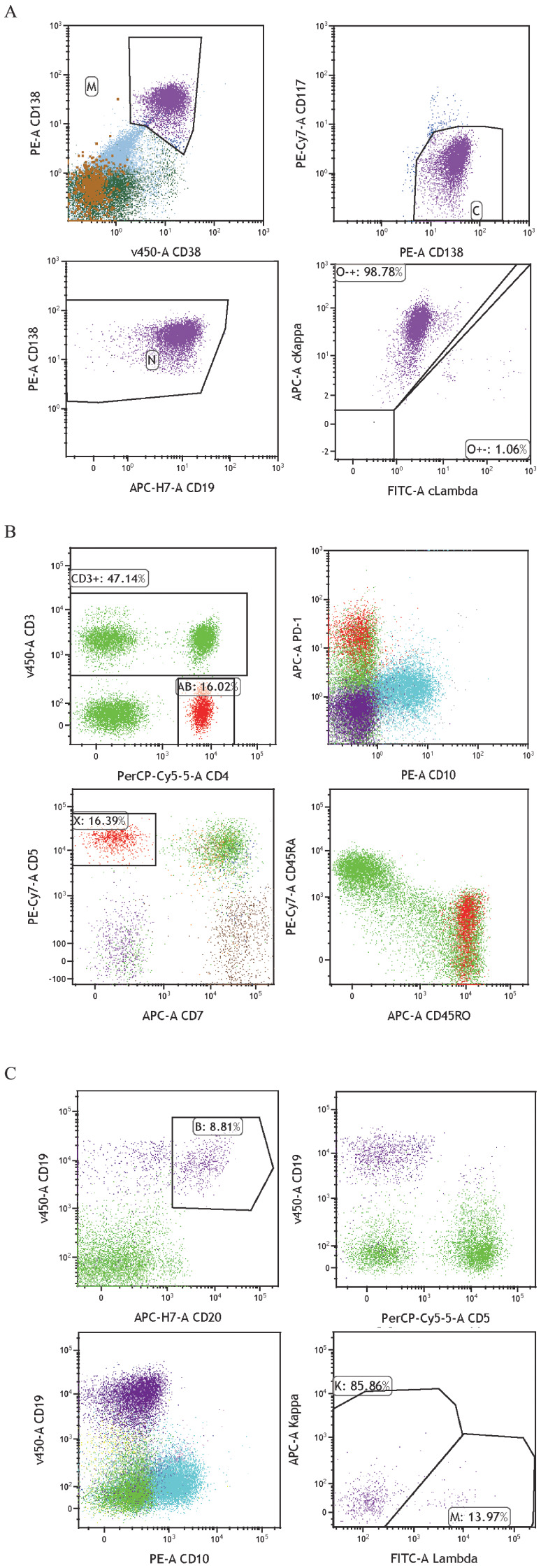
患者骨髓标本流式细胞术结果 **A** 可见一群单克隆浆细胞，表型基本正常；**B** 可见一群CD3^−^CD4^+^PD1^++^异常T淋巴细胞；**C** 可见一群CD5^−^CD10^−^单克隆B淋巴细胞

患者外院淋巴结病理活检会诊结果回报：①2021年12月淋巴结切检：淋巴结结构基本破坏，可见残留淋巴滤泡，滤泡套区不明显，可见生发中心，免疫组化CD21染色显示部分FDC网不规则，滤泡间区增宽，小血管增生，可见胞体小至中等大，胞质中等量、透明的异型细胞增生，多围绕淋巴滤泡分布，另可见少量浆细胞、嗜酸性粒细胞、中性粒细胞及活化的大细胞散在或簇状分布。我院免疫组化：CD2（+），CD7部分（+），CD10个别（+），CXCL13较多（+），PD1弱（+），CD57（−），CD15（−），CD138浆细胞（+），κ部分浆细胞（+），λ部分浆细胞（+）；PTCL，符合AITL。②2022年4月淋巴结切检：淋巴结基本结构破坏，残存少量淋巴滤泡，滤泡间区扩大，可见一类淋巴细胞散在或围绕滤泡小片状分布，胞体小，胞质量中等，核略不规则，染色质粗。另见异常浆细胞明显增生，片状分布，可见少数活化的大细胞散在分布。本院免疫组化：CD5（+），CD4（+），CD10个别（+），BCL6少数（+），PD1弱（+），CXCL13少量（+），ICOS（−），CD8（−），浆细胞CD38（+），CD23 FDC（+），CD56（−），CyclinD1（−），考虑AITL伴单克隆浆细胞增生。

基因突变筛查结果回报，检出TNFRSF14 1p36.20突变（64.5％）、TET2 4q24突变（4.50％）、STAT3 17q21.2突变（1.10％），DNMT3A 2p23.3突变（2.60％），ARID1A 1p36.11突变（26.80％）。而AITL重现性基因突变包括TET2（66％）、RHOAG17V（45％）、DNMT3A（33％）、IDH2（25％）等，其中IDH2为AITL特征性突变。虽然本例患者未检出IDH2突变，但仍可见TET2、DNMT3A等常见重现性突变，高度提示AITL可能。

结合患者复杂病史及多样的临床表现，血清EBV-DNA水平明显升高（以B细胞、NK细胞为主），免疫组化和流式细胞术显示一群表达CXCL13、PD-1的典型TFH，且二代测序可见TET2、DNMT3A疾病相关热点突变，考虑AITL诊断明确。但本病例并非单纯AITL，该患者发病过程中出现一过性急性肾衰竭、大量蛋白尿，肾脏穿刺病理考虑不除外LCPT，多次骨髓穿刺涂片示大量浆细胞增生伴淋巴样浆细胞，且淋巴结及骨髓流式细胞术可见一群单克隆浆细胞，仍需考虑多病共存可能。既往病例报道，AITL可合并多克隆浆细胞增多症和多克隆高丙种球蛋白血症，且浆细胞比例可非常高，甚至可掩盖潜在的T细胞肿瘤。对于这类少见的AITL，应与浆细胞肿瘤仔细鉴别，免疫固定电泳阴性/轻链非限制性表达/持续表达CD45可能是鉴别的关键点。该患者多次化验可见血、尿异常M蛋白（κ轻链），出现一过性急性肾衰竭，骨髓流式细胞术可见浆细胞限制性表达cκ，但浆细胞免疫表型则大致正常，回顾相关文献报道，极少数AITL患者可出现单克隆浆细胞增生，预后欠佳。同时该患者骨髓流式细胞术可见少量CD5^−^CD10^−^单克隆B细胞，伴大量EBV扩增，考虑本例患者起病于EBV感染驱动的B免疫母细胞活化增殖并形成异常免疫微环境，促进AITL发生发展，并可能进一步促进浆细胞分化及单克隆增生。最终该患者确诊为AITL伴单克隆B细胞、浆细胞增生。

## 第三次临床讨论

AITL属于PTCL的一种少见亚型，无论是单药还是强化联合治疗，缓解期均较短暂，预后欠佳，尚无金标准治疗方案，目前多推荐一线进入临床试验，或以CHOP方案为基础的诱导治疗序贯自体造血干细胞移植。研究发现表观遗传调控在PTCL发病机制中起重要作用，联用组蛋白去乙酰化酶抑制剂（HDACi）和去甲基化药物治疗PTCL疗效较好，且安全性良好。回顾既往研究，AITL合并浆细胞疾病患者较为罕见，预后极差，中位无进展生存期为4.8个月，中位总生存期仅1.7年。但患者体能状态较差，无法耐受高强度化疗，综合考虑建议患者首先应用较为温和、骨髓抑制程度较轻的双表观遗传学方案（西达本胺+阿扎胞苷）治疗AITL，并联合CD20单克隆抗体清除伴EBV异常扩增的单克隆B细胞，待血象恢复及EBV-DNA水平下降，调整为CD38单克隆抗体清除异常克隆浆细胞。

患者入院后仍反复发热，精神欠佳，食欲差，血压持续低于90/60 mmHg（1 mmHg＝0.133 kPa），胸部CT可见肺部感染，不除外感染性休克，积极予以抗感染、补液升压、营养支持等对症支持治疗后症状好转，患者及家属要求返回当地医院继续治疗。后患者因经费原因于当地医院应用双表观遗传学药物+利妥昔单抗+硼替佐米方案治疗3个疗程，3个疗程后复查血常规示血象恢复，肌酐降至正常，血浆、B细胞、T细胞、NK细胞EBV拷贝数恢复正常，血、尿M蛋白明显下降，骨髓异常淋巴细胞消失，浆细胞明显减少，乏力、纳差较前好转，食欲欠佳仍需静脉营养支持，目前第4个疗程双表观遗传学药物+利妥昔单抗+硼替佐米治疗中。

## 总结

本例患者为中老年男性，以多发淋巴结增大起病，反复发热，伴乏力、纳差，伴大量蛋白尿，且出现一过性急性肾衰竭，多次淋巴结活检病理提示EBV驱动B免疫母细胞活化增殖，流式细胞术可见三群异常细胞，同时骨髓活检可见大量单克隆浆细胞增生，最终明确诊断为AITL伴单克隆B细胞、浆细胞增生，经双表观遗传学联合免疫治疗后疾病得到有效控制。AITL并非罕见病，但由于其不典型的临床表现及复杂的免疫微环境，确诊常存在一定困难，同时本例患者出现急性肾衰竭，骨髓可见大量单克隆浆细胞增生，出现浆细胞肿瘤典型表现，极具迷惑性，诊治过程较曲折、复杂，值得共同学习。在临床工作中，医务人员应首先秉承“一元论”，避免误诊，但在少数情况下可能会出现某一种疾病无法解释所有临床表现及化验检查结果，应考虑同时合并两种疾病的可能，以避免漏诊。当前新药蓬勃发展，表观遗传学治疗、免疫治疗等已成为血液系统恶性肿瘤患者重要的治疗选择，全面评估患者体能状态并谨慎选择治疗方案可有效改善患者预后。

